# Supplementary material for: Mechanisms governing the pioneering and redistribution capabilities of the non-classical pioneer PU.1
Source: Nat Commun. 2020 Jan 21;11:402. doi: 10.1038/s41467-019-13960-2 (PMC6972792; doi:10.1038/s41467-019-13960-2)
Supplement: Supplementary file 7 — Source data [file 41467_2019_13960_MOESM7_ESM.zip › Source_Data/FigureS2/FigureS2B_MotifScanOutput/KmeansCluster4/homerResults/motif36.similar.html]

motif36

## Information for motif36

C
T
A
G
G
T
A
C
A
G
T
C
C
G
T
A
A
G
C
T
C
T
A
G
A
G
C
T
A
C
T
G
C
G
T
A
T
C
G
A
T
C
A
G
  
Reverse Opposite:  

A
G
T
C
A
G
C
T
C
G
A
T
G
T
A
C
C
T
G
A
G
A
T
C
C
T
G
A
G
C
A
T
C
T
A
G
A
C
T
G
A
G
T
C
  

|  |  |
| --- | --- |
| p-value: | 1e-23 |
| log p-value: | -5.506e+01 |
| Information Content per bp: | 1.794 |
| Number of Target Sequences with motif | 104.0 |
| Percentage of Target Sequences with motif | 2.30% |
| Number of Background Sequences with motif | 257.6 |
| Percentage of Background Sequences with motif | 0.62% |
| Average Position of motif in Targets | 91.6 +/- 46.7bp |
| Average Position of motif in Background | 98.0 +/- 70.6bp |
| Strand Bias (log2 ratio + to - strand density) | -0.3 |
| Multiplicity (# of sites on avg that occur together) | 1.02 |
| Motif File: | file (matrix) reverse opposite |

### Similar de novo motifs found

|  |  |  |  |  |  |  |  |
| --- | --- | --- | --- | --- | --- | --- | --- |
| Rank | Match Score | Redundant Motif | P-value | log P-value | % of Targets | % of Background | Motif file |
| 1 | 0.815 | C A G T A G C T G T A C A C G T A C G T G T A C C T G A A G T C C G T A C G T A A C T G A T C G | 1e-23 | -54.963327 | 1.09% | 0.11% | motif file (matrix) |
| 2 | 0.797 | T G A C G C A T A G C T A G T C A C G T G A C T G A T C C G T A A G T C C G T A G C T A A C T G A C T G | 1e-19 | -46.024770 | 0.89% | 0.09% | motif file (matrix) |
